# Gallbladder torsion with gangrenous cholecystitis: a case report

**DOI:** 10.1093/jscr/rjad252

**Published:** 2023-05-13

**Authors:** Tessa Daly, Jim Byrne, Fuad Aftab

**Affiliations:** Department of General Surgery, Cork University Hospital, Wilton Road, Cork, Ireland; Department of General Surgery, Cork University Hospital, Wilton Road, Cork, Ireland; Department of General Surgery, Mallow General Hospital, Limerick Road, Cork, Ireland; Department of General Surgery, Cork University Hospital, Wilton Road, Cork, Ireland; Department of General Surgery, Mallow General Hospital, Limerick Road, Cork, Ireland

## Abstract

A woman in her nineties presented with a 7-day history of right upper quadrant pain, nausea and anorexia. Examination findings included tenderness in the right upper quadrant and a positive Murphy’s sign. Laboratory studies were unremarkable with normal white cell count, C-reactive protein and liver functions tests. Ultrasound of the abdomen, however, demonstrated a distended gallbladder containing sludge and a thickened gallbladder wall. The patient’s symptoms worsened with associated elevation of inflammatory markers despite initial treatment with intravenous antibiotics for 24 h. It was decided to proceed to laparoscopic cholecystectomy. Intraoperatively, 360° clockwise torsion of a gangrenous gallbladder on an elongated mesentery was noted. Laparoscopic cholecystectomy was completed without complication and the patient was discharged home after completion of a course of intravenous antibiotics. Histopathological examination demonstrated acute cholecystitis with extensive mural necrosis.

## INTRODUCTION

Gallbladder torsion is a rare acute surgical disorder with a significant risk of mortality, particularly in elderly patients. Diagnosis is challenging and is only made intraoperatively in most cases. Early operative intervention may reduce the significant morbidity and mortality associated with the condition.

We report a case of complete torsion of the gallbladder on an elongated mesentery and resultant necrosis of the gallbladder in an elderly female patient. Early laparoscopic cholecystectomy allowed successful management in this case and the patient experienced an uncomplicated recovery.

### CASE REPORT

A woman in her nineties presented to the emergency department with a 7-day history of intermittently severe right upper quadrant pain with associated nausea and bloating. She had not experienced vomiting, fever or signs of obstructive jaundice. Her past medical history was significant for an open appendicectomy in childhood with a later laparotomy and adhesiolysis for small bowel obstruction. She had experienced a lumbar vertebral fracture after a fall ⁓4 months prior to presentation.

On examination, the patient was anicteric, afebrile and normotensive with heart rate 90 beats per minute. Abdominal examination revealed a palpable gallbladder with tenderness in the right upper quadrant and a positive Murphy’s sign.

Laboratory investigation completed at presentation were unremarkable with a normal white cell count (8.1 × 10^9^/L) and C-reactive protein (0.8 mg/L). All liver function tests including total bilirubin and alkaline phosphatase were within normal limits.

**Figure 1:**
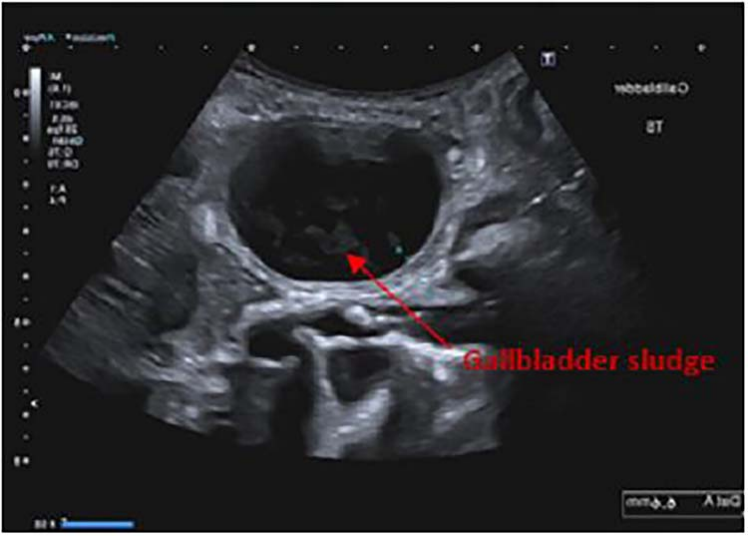
Ultrasound abdomen showing gallbladder sludge.

**Figure 2:**
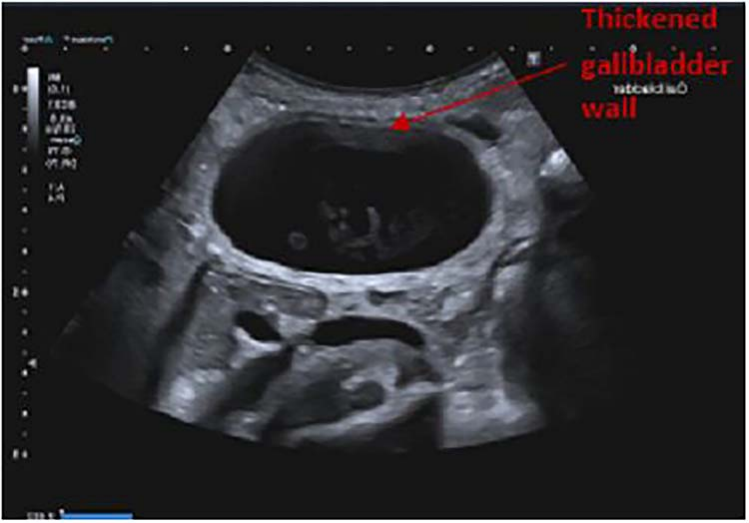
Ultrasound abdomen showing a thickened gallbladder wall.

Findings on abdominal ultrasound included a distended gallbladder with thickening of the gallbladder wall up to 7 mm. Definite cholelithiasis was not noted but the gallbladder contained sludge. No dilatation of the intrahepatic or extrahepatic biliary tree was identified (Figs 1 and 2).

She was admitted with a presumptive diagnosis of acute calculous cholecystitis. Biliary colic was also included in the differential diagnosis as inflammatory markers were not elevated but was considered less likely due to the duration of the patient’s symptoms and presence of a positive Murphy’s sign.

Non-operative treatment of acute cholecystitis with intravenous fluids, analgesia, antiemetics and intravenous antibiotics was initially trialled for this patient. Her condition deteriorated despite 24 h of conservative management with worsening abdominal pain, onset of vomiting and development of guarding in the right upper quadrant. Additionally, repeat laboratory investigations demonstrated an elevated C-reactive protein of 98.2 mg/L although white cell count remained within the normal reference range.

Given the patient’s worsening symptoms and laboratory evidence of an increasing inflammatory response, the decision was made to proceed to laparoscopic cholecystectomy after discussion of treatment options with the patient.

Intraoperatively, torsion of a gangrenous gallbladder was noted with 360° clockwise rotation of the gallbladder and resultant obstruction of the cystic duct. After detorsion of the gallbladder, an elongated and narrowed mesentery suspending the gallbladder from the gallbladder fossa was noted. Laparoscopic needle decompression of the gallbladder was completed with aspiration of haemorrhagic fluid. Cholecystectomy was successfully completed laparoscopically, and a 20 Fr Robinson drain was placed in the subhepatic space.

The patient’s postoperative recovery was uncomplicated. She completed a course of intravenous antibiotics and was discharged home after removal of the abdominal drain on postoperative day eight.

Histopathological examination of the patient’s gallbladder demonstrated acute cholecystitis with wall thickness up to 10 mm, extensive mural ischaemic necrosis with accompanying haemorrhage and areas of suppurative inflammation.

## DISCUSSION

Gallbladder torsion is defined as rotation of the gallbladder on its mesentery along the axis of the cystic duct and artery [1]. Torsion may occur in a clockwise or anticlockwise direction and can be classified as incomplete when rotation is ˂180° or complete when rotation greater than 180° occurs [2]. Complete torsion may result in obstruction of gallbladder emptying and arterial blood supply with resultant ischaemia and necrosis [1]. It is an exceedingly uncommon acute surgical disorder with ⁓300 cases reported since it was first described by Wendel in 1898 [3, 4]. Torsion of the gallbladder occurs most commonly in elderly females with a female:male ratio of 4:1 and a mean age of 64 [3].

The aetiology of gallbladder torsion is unclear but is hypothesized to occur secondary to anatomical anomalies resulting in a ‘floating gallbladder’. This anomaly is characterized by either an elongated mesentery supporting the gallbladder and cystic duct or an incomplete mesentery supporting the cystic duct only; resulting in an abnormally mobile gallbladder which is prone to volvulus [5, 6]. The existence of gallbladder torsion in children suggests congenital abnormalities of the gallbladder mesentery may be sufficient for volvulus to occur [7, 8]. Loss of visceral fat, liver atrophy and visceroptosis have also been suggested to predispose to the condition and may account for its occurrence predominantly in elderly patients [9, 10]. Gallstones are not considered a causative factor as they are only identified in one-quarter of patients with the condition [11].

The clinical and radiological features of gallbladder torsion may mimic those of acute cholecystitis and preoperative diagnosis is made on only 26% of cases [3]. Lau *et al*. proposed three triads of features of gallbladder torsion that may raise clinical suspicion of the condition. These include patient features such as a thin, elderly patient with chronic respiratory disease or spinal deformity, symptoms including short duration of symptoms, early onset of vomiting and abdominal pain as well as physical signs such absence of jaundice, lack of toxaemia and tachycardia in the absence of pyrexia [12]. Nakao *et al.* also suggest that acute onset, lack of fever or jaundice, enlargement of the gallbladder and poor response to antibiotic therapy may aid in differentiating torsion of the gallbladder from acute cholecystitis [11].

Management of gallbladder torsion consists of prompt decompression and detorsion of the gallbladder to allow visualization of the cystic duct and artery followed by cholecystectomy. Laparoscopic cholecystectomy is the preferred approach to treatment and in many cases diagnosis of gallbladder torsion is only made at time of laparoscopy [3, 13].

Prompt treatment of gallbladder torsion is required to prevent ischaemia and perforation of the gallbladder that increases morbidity and mortality, particularly in the elderly population predominantly affected by this condition. Overall mortality rates for torsion of the gallbladder have been reported between 4.9 and 6% [3, 11].

## Data Availability

All de-identified participant data that underlie the results reported in the article will be made available. Data will be available beginning immediately following publication with no end date. It will be made available to anyone who wishes to access the data for any purpose. Proposals should be submitted to 116306691@umail.ucc.ie.
